# Search for thermodynamically stable ambient-pressure superconducting hydrides in the GNoME database

**DOI:** 10.1038/s42005-026-02552-4

**Published:** 2026-02-27

**Authors:** Antonio Sanna, Tiago F. T. Cerqueira, Ekin Dogus Cubuk, Ion Errea, Yue-Wen Fang

**Affiliations:** 1 Max-Planck-Institut für Mikrostrukturphysik, Weinberg 2, D-06120 Halle, Germany; 2 Institut für Physik, Martin-Luther-Universität Halle-Wittenberg, D-06099 Halle, Germany; 3 CFisUC, Department of Physics, University of Coimbra, Rua Larga, 3004-516 Coimbra, Portugal; 4 Periodic Labs, San Francisco, CA USA; 5 Fisika Aplikatua Saila, Gipuzkoako Ingeniaritza Eskola, University of the Basque Country (UPV/EHU), Europa Plaza 1, 20018 Donostia/San Sebastián, Spain; 6 Centro de Física de Materiales (CFM-MPC), CSIC-UPV/EHU, Manuel de Lardizabal Pasealekua 5, 20018 Donostia/San Sebastián, Spain; 7 Donostia International Physics Center (DIPC), Manuel de Lardizabal Pasealekua 4, 20018 Donostia/San Sebastián, Spain

**Keywords:** Superconducting properties and materials, Theory and computation

## Abstract

Hydrides are considered to be one of the most promising families of compounds for achieving high temperature superconductivity. However, there are very few experimental reports of ambient-pressure hydride superconductivity, and the superconducting critical temperatures (*T*_c_) are typically less than 10 K. At the same time several hydrides have been predicted to exhibit superconductivity around 100 K at ambient pressure but in thermodynamically unfavorable phases. In this work we aim at assessing the superconducting properties of thermodynamically stable hydride superconductors at room pressure by investigating the GNoME material database, which has been recently released and includes thousands of hydrides thermodynamically stable at 0K. To scan this large material space we have adopted a multi stage approach which combines machine learning for a fast initial evaluation and cutting edge ab initio methods to obtain a reliable estimation of *T*_c_. Ultimately we have identified 25 cubic hydrides with *T*_c_ above 4.2 K and reach a maximum *T*_c_ of 17 K. While these critical temperatures are modest in comparison to some recent predictions, the systems where they are found, being stable, are likely to be experimentally accessible and of potential technological relevance.

## Abstract (web summary)

The recently published GNoME material database contains a large number of new thermodynamically stable hydride compounds. In this work the authors assess their superconducting properties using ab initio methods and identify 25 cubic hydrides with a maximum critical temperature of 17 K.

## Introduction

The near room temperature superconductivity, experimentally observed in several superconducting hydrides such as LaH_10_^1,2^ and YH_9_^3^ hosting superconductivity above −30 and −23 °C, respectively, demonstrates that the investigation of phonon-mediated superconductors involving the lightest element H is well merited^4^. However, achieving near room temperature superconductivity in these hydrides requires subjecting them to extreme pressures over 170 billion pascals (170 GPa), making the real-world application unlikely.

Given the tough conditions of these high-*T*_c_ superconducting hydrides posed by the high pressures, one may naturally wonder whether it is possible to achieve superconductivity in the hydrides at ambient pressure^5^. In earlier studies, very few hydride superconductors have been experimentally reported at ambient pressure, and their superconducting transition temperatures are typically less than 10 K^6–8^. The desire to discover high-*T*_c_ ambient-pressure superconducting hydrides has driven some ab initio theoretical predictions recently^9–16^. In particular, Cerqueira et al. combined the machine learning methods with the ab initio study to predict around 50 superconducting hydrides with *T*_c_ above 20 K from the screening of more than 1 million compounds^9^. The cubic hydride family with *M*H_6_ octahedra is exceptionally attractive because some of them are near the convex hull and can maintain a high *T*_c_ above the boiling point of liquid nitrogen of 77 K^10,11^. Several perovskite hydrides have also attracted some attention due to their high *T*_c_ and small enthalpy distance from the convex hull^9,17,18^. Remarkably, ab initio calculations suggest that RbPH_3_ is dynamically and kinetically stable at ambient pressure with a superconducting critical temperature of about 100 K^19^.

As discussed in refs. ^20^ and ^15^, the evidence that emerges from recent high-throughput research is that high-*T*_c_ values of the order of 100 K appear to be systematically linked to some degree of thermodynamical instability. This may be attributed to the destabilizing effect of delocalized hydrogen-derived electrons in the metallic bonding environment of multinary hydrides. Consequently, the hydrides thermodynamically stable at 0K in large materials databases such as Materials Project are generally semiconductors or insulators. Clearly, this drops a large fraction of the problem to the synthesis, as one expects that a synthesis route for metastable compounds might be very hard to find, although some recent advances in pressure-quench protocols suggest that it may be possible to retain superconductivity in the ambient-pressure metastable phases quenched from a pressure where the corresponding phases are thermodynamically stable^21^.

GNoME has used active learning and graph neural networks to identify 381 thousand crystals that are stable at 0 K according to DFT^22^. Most of these structures are identified by elemental substitutions, a commonly used method to search for 0 K stable crystals^23–25^. As mentioned in the conclusion of GNoME, these calculations are not able to consider configurational disorder. With further improvements in force fields and computational resources, simulating finite temperature stability in a high-throughput manner might be possible. Given this set of crystals that are stable at 0 K according to DFT, might there be interesting superconducting candidates? In this work, we investigate this possibility by means of first-principles simulations.

A brute force use of ab initio methods^26^ would be computationally too expensive to be directly applicable to such a large space of materials. For this reason, we adopt a recently developed machine-learning accelerated ab initio algorithm. This allows us to identify possible high-*T*_c_ hydrides in the GNoME database and use high-level ab initio methods to validate machine learning predictions. As we will report below, the search has been successful in identifying 25 cubic hydrides with *T*_c_ exceeding the boiling point of liquid helium (4.2 K). Most of these hydrides have *T*_c_ lower than NbTi alloys (10 K), nevertheless the cubic LiZrH_6_Ru hydride with vacancy-ordered double perovskite structure stands out and shows a *T*_c_ at 17 K at ambient pressure.

## Results

### Machine learning accelerated a high-throughput ab initio approach

**Fig. 1:**
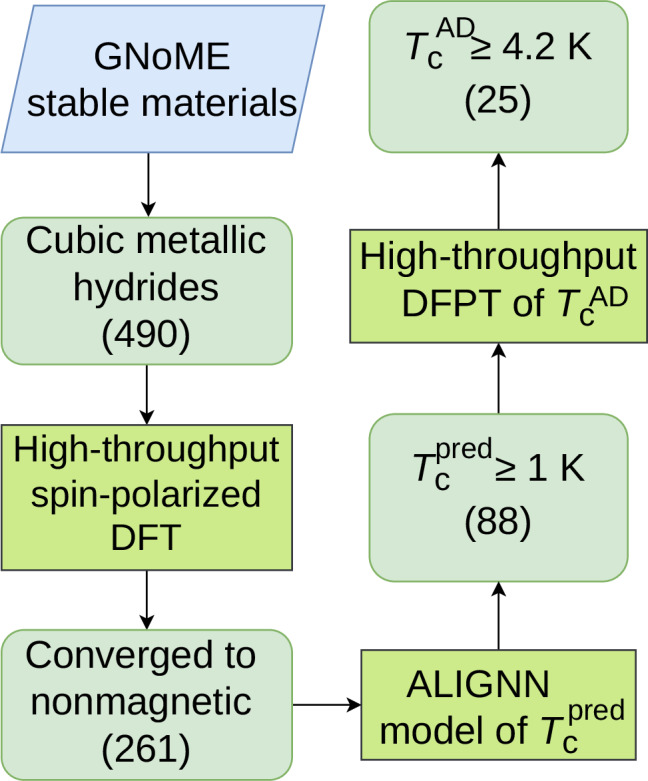
The computational workflow of the screening of cubic superconducting hydrides with thermodynamic stability by combining the ab initio study with the ALIGNN model. The integers in parentheses indicate the number of hydrides.

To accelerate the discovery of superconducting hydrides, we have combined the machine learning methods with high-throughput ab initio calculations. The major workflow of the superconductors screening is described in Fig. 1.

We first performed data mining on thermodynamically stable hydrogen-based compounds at 0K with a cubic structure and metallic properties from the openly available dataset of GNoME project^27^, which resulted in a subset including 490 hydrides. We focused exclusively on cubic hydrides because the cubic structure is a recurring and well-established structural motif across many classes of high-*T*_c_ hydrogen-based compounds at both high pressure (e.g., LaH_10_^1,2^ and YH_9_^3^) and ambient pressure (e.g., RbPH_3_^19^ and Li_2_AgH_6_^15^). High-throughput spin-polarized density functional theory (DFT) calculations were further used to find 261 nonmagnetic metallic hydrides. Then, the Atomistic Line Graph Neural Network (ALIGNN) model^28^, which has been proven successful in the discovery of superconductors^9,10^, was used to estimate *T*_c_ of the hydrides. To narrow down the search space of superconductors and reduce the computational cost of ab initio calculations, only the superconducting hydrides with ALIGNN-predicted ${T}_{{{{\rm{c}}}}}^{{{{\rm{pred}}}}}$ ≥ 1 K were considered in the subsequent high-throughput density functional perturbation theory (DFPT) calculations.

Using high-throughput DFPT methods coupled with Allen–Dynes formula, we found 25 cubic hydrides from the GNoME database with a superconducting critical temperature ${T}_{{{{\rm{c}}}}}^{\,{{{\rm{AD}}}}}$ ≥ 4.2 K (see Tables 1 and 2). For the 25 cubic hydrides, the DFPT-calculated ${T}_{{{{\rm{c}}}}}^{\,{{{\rm{AD}}}}}$ and ALIGNN-predicted ${T}_{{{{\rm{c}}}}}^{{{{\rm{pred}}}}}$ are compared in a parity plot included in Supplementary Fig. S1. The values of ALIGNN-predicted ${T}_{{{{\rm{c}}}}}^{{{{\rm{pred}}}}}$ are also included in Tables 1 and 2 explicitly. The ALIGNN predicted *T*_c_ shows a strong quantitative agreement with DFPT-calculated ${T}_{{{{\rm{c}}}}}^{\,{{{\rm{AD}}}}}$, with a mean absolute error (MAE) of only 2.5 K. Given that the MAE of 2.5 K is significantly smaller than the target benchmark (the boiling point of liquid helium, 4.2 K), this predictive uncertainty is acceptable for pre-screening purposes. In addition, the conservative criterion of ALIGNN-predicted ${T}_{{{{\rm{c}}}}}^{{{{\rm{pred}}}}}$ ≥ 1 K should ensure that all materials with true superconducting critical temperature above 4.2 K are retained in the high-throughput DFPT calculations.

**Table 1:** Vacancy-ordered double perovskites

mat_id	formula	*λ*	${\omega }_{\log }$	${T}_{{{{\rm{c}}}}}^{\,{{{\rm{AD}}}}}$	${T}_{{{{\rm{c}}}}}^{{{{\rm{pred}}}}}$
**Vacancy-ordered double perovskites**					
a1c4087396	EuDyReH_6_	2.05	35.84	4.9	2.5
2523d9ecde	NdEuTcH_6_	0.66	166.66	5.1	3.9
1243cdd0e1	Ce_2_HRh_6_	0.67	167.00	5.1	1.6
97ff896f6f	SmEuTcH_6_	0.67	168.28	5.3	6.2
0eddd1b470	EuCdFeH_6_	0.47	502.93	5.4	4.3
cc52a3846d	EuYTcH_6_	0.67	179.97	5.6	5.7
e5427bdb32	TbEuTcH_6_	0.73	168.49	6.3	6.8
84f266aecd	EuDyTcH_6_	0.74	166.47	6.5	7.7
9248b5ab15	EuHoTcH_6_	0.77	163.21	6.9	9.1
4bb5b78a22	SmEuReH_6_	0.98	105.75	6.9	2.9
68f1be98a3	LiCeMnH_6_	0.65	234.44	6.9	2.7
5e1177e128	EuErTcH_6_	0.81	160.52	7.6	9.5
8d4721d779	EuYReH_6_	1.28	84.82	7.7	3.3
f68afdcbbc	EuTmTcH_6_	0.88	155.98	8.4	9.3
1e099eabf5	YbCeTcH_6_	0.90	150.52	8.6	6.1
1bd4b03f35	YbGdTcH_6_	1.00	144.04	9.6	10.2
8bf27d9be2	LiTiH_6_Ru	1.12	123.38	9.8	8.7
e5c2a73dc0	EuLuTcH_6_	1.09	134.38	10.1	9.2
51a97ab68d	EuCdH_6_Ru	2.53	84.40	13.6	6.7
0f97a7734c	LiZrH_6_Ru	1.00	341.59	23.5	4.7

Superconducting transition temperatures calculated with the Allen–Dynes formula (${T}_{{{{\rm{c}}}}}^{\,{{{\rm{AD}}}}}$ in K) above 4.2 K, logarithmic average of the phonon frequencies (${\omega }_{\log }$ in K), and the electron–phonon coupling constant (*λ*) for vacancy-ordered double perovskites. The ALIGNN-predicted ${T}_{{{{\rm{c}}}}}^{{{{\rm{pred}}}}}$ (K) are included in the last column for comparison with ${T}_{{{{\rm{c}}}}}^{\,{{{\rm{AD}}}}}$.

**Table 2:** Fluorite-like hydrides

mat_id	Formula	*λ*	${\omega }_{\log }$	${T}_{{{{\rm{c}}}}}^{\,{{{\rm{AD}}}}}$	${T}_{{{{\rm{c}}}}}^{{{{\rm{pred}}}}}$
*Fluorite-like*					
d8e8825efe	Ta_3_VH_8_	0.64	178.58	4.9	4.1
149cfed18b	Ta_3_NbH_8_	0.63	190.37	5.0	4.4
34fc41965b	TaNb_3_H_8_	0.72	185.95	7.0	7.1
*Vacancy-ordered fluorite-like*					
140fb04a4b	Ta_6_NbH_16_	0.64	179.61	5.0	6.0
d93c6d6bda	Ta_6_MoH_16_	0.75	176.75	7.3	6.0

Superconducting transition temperatures calculated with the Allen-Dynes formula (${T}_{{{{\rm{c}}}}}^{\,{{{\rm{AD}}}}}$ in K) above 4.2 K, logarithmic average of the phonon frequencies (${\omega }_{\log }$ in K), and the electron-phonon coupling constant (*λ*) for fluorite-like hydrides and vacancy-ordered fluorite-like hydrides. The ALIGNN-predicted ${T}_{{{{\rm{c}}}}}^{{{{\rm{pred}}}}}$ (K) are included in the last column for comparison with ${T}_{{{{\rm{c}}}}}^{\,{{{\rm{AD}}}}}$.

By examining the crystal structures of the 25 thermodynamically stable superconducting hydrides, we find that they can be grouped into two prototype structure families, i.e., vacancy-ordered double perovskites and fluorite-like structure with or without vacancy-ordering.

**Fig. 2:**
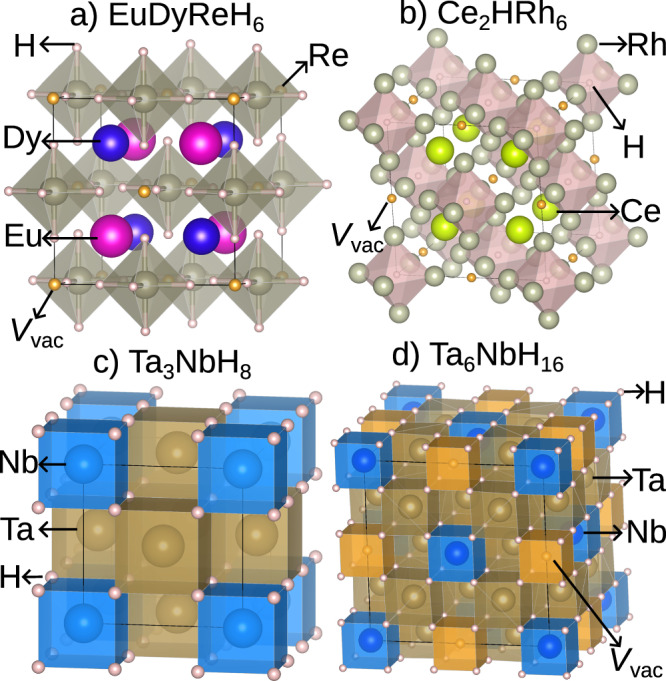
The conventional cells of the hydrides are selected from vacancy-ordered double perovskites and fluorite-like structures. **a** Normal double perovskite EuDyReH_6_ with vacancy ordering. **b** Inverted double perovskite Ce_2_HRh_6_ with vacancy ordering. **c** Fluorite-like Ta_3_NbH_8_. **d** Fluorite-like Ta_6_NbH_16_ with vacancy. The gold spheres represent the ordered vacancies *V*_vac_.

All the vacancy-ordered double perovskites can be generalized as defect forms of doubly cation-ordered $AA{\prime} BB{\prime} {X}_{6}$ (see Fig. 2a). In the defect-free $AA{\prime} BB{\prime} {X}_{6}$, *X* is stabilized at 24f Wyckoff position; *A* and $A^{\prime}$ are located at 4c and 4d Wyckoff positions, and are bonded to twelve equivalent *X* to form *AX*_12_ and $A^{\prime} {X}_{12}$ cuboctahedra; *B* and $B^{\prime}$ reside at 4a and 4b Wyckoff positions, and each *B* or $B^{\prime}$ is bonded to six equivalent *X* ions to form *BX*_6_ and $B^{\prime} {X}_{6}$ octahedra. In the hydrides with vacancy-ordered double perovskite structure, the number of atomic sites is reduced to nine per formula unit due to the existence of vacancies at either 4a or 4b Wyckoff positions. Figure 2a displays EuDyReH_6_ as an example of a vacancy-ordered double perovskite where the vacancy appears at the 4a position and hydrogen atoms are located at the *X* site. In contrast to vacancy-ordered double perovskites with *X* being H and space group being $F\bar{4}3m$ (No. 216), the hydrogen atoms are swapped with the metallic Rh atoms in Ce_2_HRh_6_, forming the inverted double perovskite with space group of $Fm\bar{3}m$ (No. 225), where 4c and 4d positions are both occupied by Ce and the vacancy is located at the 4b position. As seen in Table 1, all these vacancy-ordered double perovskites exhibit a superconducting critical temperature ranging from 4.9 K in EuDyReH_6_ to 23.5 K in LiZrH_6_Ru, and most of them show a weak electron–phonon coupling constant *λ* of less than 1. In addition, these materials show a wide range of ${\omega }_{\log }$ from 35.84 K in EuDyReH_6_ to 502.93 K in EuCdFeH_6_. The hydride EuCdH_6_Ru has the largest *λ* of 2.53, but its critical temperature remains as small as 13.6 K due to the very small ${\omega }_{\log }$ of 84.40 K.

Except for vacancy-ordered double perovskites, the remaining materials are fluorite-like (CaF_2_-like) structured. For example, in the $Pm\bar{3}m$ Ta_3_NbH_8_ with 12 atomic sites in the primitive cell, each Ta at 4c position and each Nb at 1a position are bonded in a body-centered cubic geometry to eight equivalent H atoms at the 8c site. In contrast, in some other fluorite-like hydrides, such as $Fm\bar{3}m$ Ta_6_NbH_16_, the alternative appearance of vacancy and Nb atoms is observed at the 1a site, leading to 23 atomic sites in the primitive cell. We can find from Table 2 that these fluorite-like hydrides show very similar *λ* and ${\omega }_{\log }$ with averages of 0.68 and 182 K. As a result, the calculated ${T}_{{{{\rm{c}}}}}^{\,{{{\rm{AD}}}}}$ are very close, all around 5.0–7.0 K. It is noted that $P\bar{4}3m$ LuNH also features a fluorite-like crystal structure with *T*_c_ of around 16 K and *λ* of around 0.78 at ambient pressure, despite the fact that this phase is very thermodynamically unstable–around 0.5 eV/atom above the convex hull^29,30^.

### Improved analysis of LiZrH_6_Ru using McMillan–Allen–Dynes formulas

**Fig. 3:**
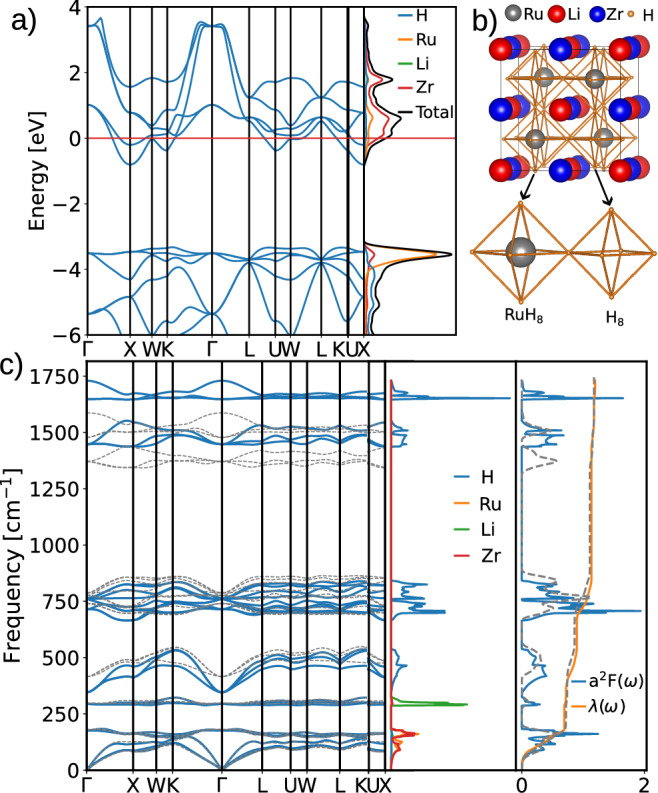
Properties of LiZrH_6_Ru at ambient pressure. Electronic structure (**a**), crystal structure (**b**), and phonon and electron–phonon coupling (**c**). The gray dashed lines in the diagrams of phonon spectrum and Eliashberg spectral functions were calculated by including the ionic quantum and anharmonic effect in the stochastic self-consistent harmonic approximation (SSCHA).

The quarterly hydride LiZrH_6_Ru has the highest ${T}_{{{{\rm{c}}}}}^{\,{{{\rm{AD}}}}}$ among the studied materials. As seen in Fig. 3a, there are two bands across the Fermi level. The Li atom is fully ionized and does not contribute to the density of states (DOS) at the Fermi level. Alternatively, the Zr atom dominates the DOS at the Fermi level, with the Ru and H atoms making minor contributions.

Because the Fermi level is located on a shoulder near the peak of the density of states, we performed the improved accuracy calculations and tested the convergence against the smearing, *k*-grid, and *q*-grid. In the improved calculations with a *k*-grid (*q*-grid) of 42^3^ (6^3^), the converged *λ* are ${\omega }_{\log }$ are 1.20 and 343.32 K, respectively. Compared to the results of the high-throughput calculations shown in Table 1 (i.e., *λ* ~1.0 and ${\omega }_{\log }$ ~341.59 K), the electron–phonon coupling constant and the logarithmic average of the phonon frequencies both increase in accurate calculations and result in a higher ${T}_{{{{\rm{c}}}}}^{\,{{{\rm{AD}}}}}$ of 30.70 K.

Since the ionic quantum and anharmonic effects could be crucial to the crystal structure, dynamical stability and the superconducting properties^31^, we have further fully relaxed the crystal structure with the stochastic self-consistent harmonic approximation (SSCHA) method^32–35^. Compared to the structure obtained in the standard DFT of Quantum Espresso, the lattice constants are unchanged (*a* = 4.604 Å, *α* = 60° in the primitive cell), and the atomic positions of the metal ions are preserved by the symmetry. However, the hydrogen positions are further optimized in SSCHA due to the ioinc quantum and anharmonic effect. Because only the hydrogen positions are slightly different in the two crystal structures, we only show the fully relaxed crystal structure from SSCHA calculations in Fig. 3b. As clearly seen by the zoom-in inset in Fig. 3b, there are two different types of octahedra, i.e., RuH_8_ and H_8_. The volume of the RuH8 octahedron is larger than that of the H8 octahedron because the central Ru atom in the RuH8 octahedron has a large atomic radius, whereas the H8 octahedron contains a vacant site at its center. Compared to the crystal structure in harmonic approximation, although every hydrogen octahedron remains perfect without showing any distortion in the SSCHA structure, the edges in H_8_ are slightly decreased from 2.178 Å in harmonic approximation to 2.160 Å in SSCHA. On the other hand, the edges in RuH_8_ are slightly increased from 2.426 Å in harmonic approximation to 2.444 Å in SSCHA. The optimizations of the hydrogen atoms in the H_8_ and RuH_8_ octahedra are also implied in the phonon dispersions in Fig. 3c, in which the whole high-frequency region of the pure hydrogen modes above 1250 cm^−1^ is softened by the anharmonicity renormalization. Despite the large downshift in the phonon energy, the accumulated electron-phonon coupling is not significantly changed according to the diagram of Eliashberg spectral functions in Fig. 3c. Consequently, there is only a mild improvement in ${T}_{{{{\rm{c}}}}}^{\,{{{\rm{AD}}}}}$, being 32.0 K with quantum effect.

**Fig. 4:**
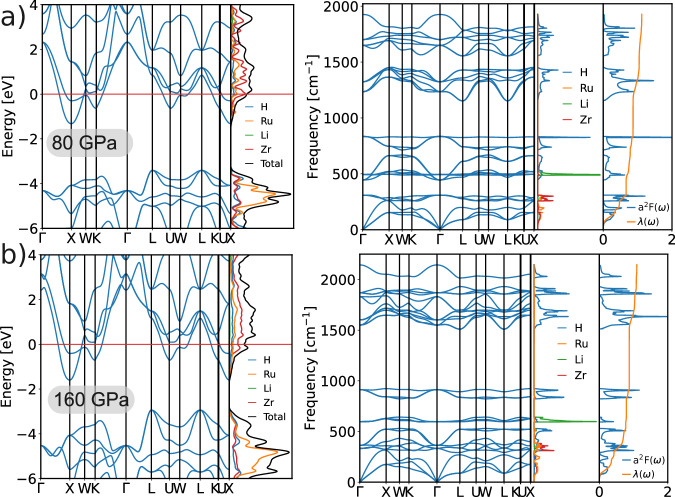
Electronic, phonon, and electron–phonon coupling properties of LiZrH_6_Ru. Top panels **a** refer to 80 GPa and bottom panels **b** to 160 GPa. In either panel **a** or **b**, the left plot shows the electronic band structure and the atom projected density of states, and the right plot displays the phonon band structure, the atom projected phonon density of states, the electron-phonon spectral function *α*^2^*F*(*ω*) and its integration curve *λ*(*ω*).

We also studied the effect of pressure on LiZrH_6_Ru in the range from 20 to 200 GPa. LiZrH_6_Ru is found to be dynamically stable up to 180 GPa in the harmonic approximation and becomes dynamically unstable at 200 GPa due to the appearance of an imaginary mode at the L point (see Supplementary Fig. S2). Our electron-phonon coupling calculations show that the superconducting temperature ${T}_{{{{\rm{c}}}}}^{\,{{{\rm{AD}}}}}$ can be enhanced at pressures up to 180 GPa compared to that at ambient pressure. In particular, the ${T}_{{{{\rm{c}}}}}^{\,{{{\rm{AD}}}}}$ is boosted to 44 K at 80 GPa. A more specific evolution of ${T}_{{{{\rm{c}}}}}^{\,{{{\rm{AD}}}}}$ and *λ* up to 180 GPa can be found in the Supplementary Fig. S3. In Fig. 4, we show the electronic, phonon, and electron–phonon coupling properties at 80 and 160 GPa. By comparing the electronic bands at pressures of 80 and 160 GPa with those at ambient pressure (i.e., Fig. 3), the bands become more dispersive with the increase in pressure. This is because the pressure makes the atoms closer, enhancing the hopping integral between neighboring orbitals and increasing the band width. As seen from the DOS diagrams in Figs. 3 and 4, the DOS at the Fermi level is mostly contributed by Zr at all pressures. We find that only at 80GPa the Fermi level lies at the sharp peak of the projected DOS of Zr, while the Fermi level is located at the shoulder of the DOS at other pressures. In addition, the diagrams of electron-phonon spectral functions *α*^2^*F*(*ω*) (right plots in Figs. 3 and 4) find that the *λ* value is 1.22 at 80 GPa that is higher than *λ* of 1.19 at 0 GPa and *λ* of 1.11 at 160 GPa. Consequently, the combination of high DOS at the Fermi level with the large *λ* at 80 GPa is responsible for the highest ${T}_{{{{\rm{c}}}}}^{\,{{{\rm{AD}}}}}$ at this pressure among the studied pressures.

### In-depth characterization of LiZrH_6_Ru beyond McMillan–Allen–Dynes formulas

So far, we have estimated the superconducting properties using the McMillan–Allen–Dynes formulas in the μ* approximation and assuming a conventional repulsion strength μ^*^ = 0.1. This approach is usually reliable when dealing with conventional superconductors^26,36^, however, one should consider that it has been developed using data from simple superconducting systems, and it is known to fail in describing systems with complex electronic properties, e.g., Magnesium diboride^37,38^.

LiZrH_6_Ru has several features that hint at the possibility that the McMillan estimation of *T*_c_ might not be very reliable. The first thing is that the Fermi level is located close to a peak in the density of states, but close to a band gap. This might lead to a poor Coulomb renormalization^39–41^ and an overestimation of *T*_c_. In simple terms this point can be explained by using the Morel-Anderson formula^42^, which links μ^*^ to the Coulomb repulsion at the Fermi level (*μ*):

1 $${\mu }^{* }=\frac{\mu }{1+\mu \,{{{\rm{ln}}}}\left(\frac{{E}_{F}}{{\omega }_{c}}\right)},$$

where *ω*_*c*_ is a cutoff frequency (arbitrary in Eliashberg theory, close to the maximum frequency of the phonon spectrum in McMillan–Allen–Dynes theory) and *E*_*F*_ is the Fermi energy setting the bandwidth of the valence region. In LiZrH_6_Ru, the valence band starts about 10 eV below the Fermi level; however, the presence of a band gap between −1 and −3 eV complicates the matter. In practice, it is not obvious if one should assume *E*_*F*_ = 1 or *E*_*F*_ = 10. In fact, inspecting the character of the electronic bands, one can see that bands close to the Fermi level are almost exclusively transition metal states, while low-lying states have a dominant H contribution, which is small at the Fermi level.

**Fig. 5:**
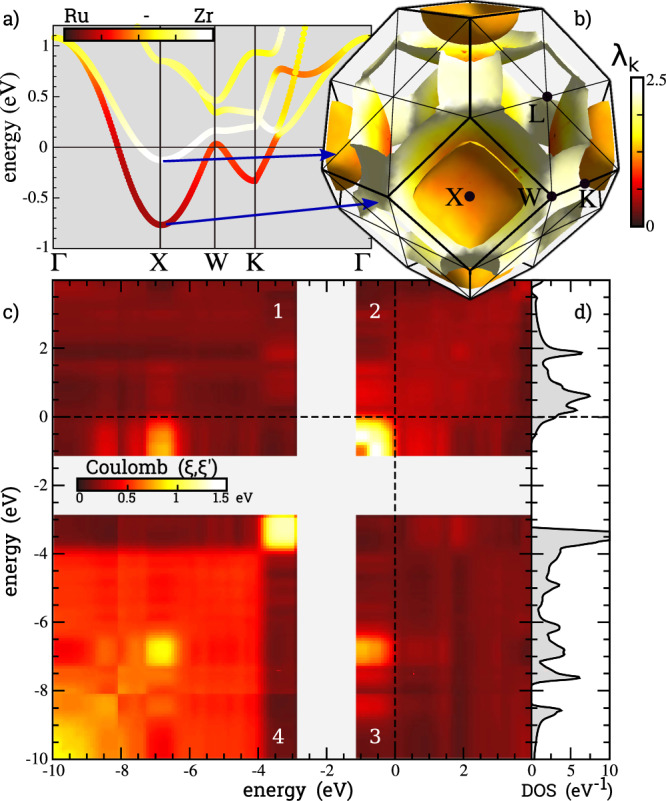
Coupling properties of LiZrH_6_Ru. **a** Electronic bands near the Fermi level, including atomic projections on the Zr and Ru sites. **b** Fermi surface and the electron-phonon coupling (*λ*_k_). **c** Coulomb interaction matrix resolved on iso-energy surfaces. **d** Density of electronic states.

Second, we see that the Fermi level states have a strong Ru and a Zr component. It is not uncommon that superconductivity in transition metals is affected by incipient magnetism. For example, the critical temperature of Nb (right next to Zr in the periodic table) is significantly reduced by the effect of spin fluctuations^43–45^. Last but not least, as shown in Fig. 5a, there are two electronic bands crossing the Fermi level with different orbital character. Multi-band effect in superconductivity might lead to an increase in *T*_c_ as compared to a single band approximation^38,46–48^. The **k**-resolved electron–phonon coupling (*λ*_**k**_) on the Fermi surface is shown^49^ in Fig. 5, showing a substantial coupling anisotropy, although neither as strong or simple as in MgB_2_.

The first point can be addressed by computing the critical temperature by solving the Eliashberg equations, including the Coulomb interaction from first principles^50,51^. This approach includes the full electronic bandwidth of the problem and the dielectric screening in the random phase approximation. The Coulomb matrix is shown in Fig. 5. This 2D matrix ($W(\xi ,\xi ^{\prime} )$) is defined by averaging the Coulomb matrix elements between iso-energetic electronic states^40,45^. By definition, *μ* = *W*(0, 0)*N*_*F*_, where *N*_*F*_ is the DOS at the Fermi level. In the random phase approximation (RPA), we have *μ* = 0.66, which is quite large, but not uncommon in transition metal compounds^45^. As one can see, this matrix has approximately a block diagonal structure. The main valence states (panel c4) are strongly repulsive in the −10 to −4 eV range, but they interact weakly with the Fermi level states (panels c3 and c1). This fact, the large value of *μ* and the presence of a large band gap, lead to an extremely disruptive Coulomb contribution to the superconducting state. The calculated *T*_c_ solving the isotropic Eliashberg in RPA is 14.2 K. This estimation is about half of that obtained by the standard McMillan–Allen–Dynes approach, an uncommon deviation which reminds the case of Chevrel phases discussed in ref. ^41^.

A second aspect we would like to further investigate is the possible role of magnetic effects in superconductivity. LiZrH_6_Ru is non-magnetic in the sense that we could not stabilize any ferromagnetic or antiferromagnetic ground state order. However, transition metals might have an incipient tendency toward magnetism, which results in a large spin susceptibility, which enhances the Coulomb repulsion. In extreme cases, this might lead to unconventional superconductivity^52,53^ and in more conventional cases to a reduced value of *T*_c_^43^. This can be included in our ab initio Eliashberg formalism using the Kukkonen-Overhauser approach discussed in refs. ^45,54^. This leads to a *T*_c_ estimation of 11.3 K. Considering that, the ab initio inclusion of the spin susceptibility tends to give a stronger Coulomb repulsion even in the absence of significant magnetic instabilities, which is consistent with a marginal role of magnetic effects.

The final aspect we wish to address is the possibility of multiband / anisotropic superconductivity. With this motive, we have computed the two-band resolved *α*^2^*F* function and solved the multiband Eliashberg equation. This approach gives an enhanced *T*_c_ of about 20%. A nearly identical enhancement was computed using the fully anisotropic superconducting density functional theory (SCDFT) approach^55^, proving that the two-band effect is the leading contribution to the anisotropic enhancement of *T*_c_. As shown in Fig. 5a, the two portions of the Fermi surface are chemically different and spatially disjoint. The inner Fermi surface has a mix of Ru–Zr character, while the outer one has a pure Zr projection. The components of the electron-phonon coupling from the inner and outer bands are *λ* ~ 0.84 and *λ* ~ 0.3, respectively. While these values alone could suggest strong anisotropy effects, similar to the case of MgB_2_, unlike MgB_2_, the inter-band coupling is also very large, scattering from the outer band to the inner one has *λ* = 0.86. In this situation, the band anisotropy is washed out by the electron-phonon coupling, leading to a minor anisotropy contribution to *T*_c_. In the language of the Suhl, Matthias, Walker paper^46^, anisotropy has a small effect on *T*_c_ because the maximum eigenvalue of the *λ* matrix (1.32) is close to the isotropic coupling (1.26). Our best estimation of *T*_c_, including multiband effects and the ab-initio RPA Coulomb interaction, is 17 K.

It is natural to compare this relatively low *T*_c_ predicted for LiZrH_6_Ru and those predicted for a similar family of compounds, Mg_2_*M*H_6_ (*M* = Rh, Ir, Pd, or Pt) in ref. ^10^ and refs. ^11,14^. The key difference is that in the Mg_2_*M*H_6_ family, hydrogenic states are present at the Fermi level and provide a significant contribution to the superconducting coupling. On the other hand, in the LiZrH6Ru system, the hydrogen contribution is negligible on the Fermi surface. A generally identical situation has been discussed in detail in ref. ^56^ to explain the different superconducting properties of Mg_2_IrH_6_ and Ca_2_IrH_6_. The fact that the Mg_2_*M*H_6_ family has high *T*_c_ but is thermodynamically unstable while LiZrH_6_Ru is thermodynamically stable is consistent with the recent analysis reported in ref. ^15^ about the limits of *T*_c_ in conventional superconductors. On a positive note, the synthesis of LiZrH_6_Ru might be easier owing to the favorable thermodynamics, while that of Mg_2_IrH_6_ is expected to be challenging^57^.

### A note about ab initio predictions and their reliability

The predictive power of computational methods in superconductivity research has evolved substantially over the past decades; however, several challenging limitations remain that may affect the confidence of ab initio predictions. Therefore, it is important to discuss what we consider to be the main sources of uncertainty that may lead to discrepancies between computational predictions and experimental realizations.

The first major source of uncertainty lies in the identification of thermodynamically stable phases. The typical error in energy estimations, while strongly material dependent, is of the order of 50 meV atom^−1^^58^. Therefore, if the energy cost for a thermodynamic decomposition is less than this, theoretical predictions should be considered extremely uncertain.

A second source of uncertainty derives from the fact that, even if a phase is truly thermodynamically stable, its synthesis might not achieve a sufficiently pure form so that predictions for a perfect bulk phase still apply. This is mostly a matter of experimental work, when superconductors are of great technological interest (e.g., high temperature superconductors), synthesis methods are investigated with utmost care to achieve samples of the highest quality. In other cases, there might not be a sufficient motivation to invest resources in optimizing the synthesis process. This consideration also applies to metastable phases, which may be accessible only through complex synthesis pathways and are therefore worth investigating primarily when a compound is expected to exhibit exceptional properties. This may be the case for Mg_2_IrH_6_, which is predicted to have an extremely high Tc^9,10^; however, its slight metastability led, upon attempted synthesis, to the formation of Mg_2_IrH_5_, which is unfortunately a semiconductor^57^.

A third source of uncertainty stems from the theory of superconductivity itself and from the state-of-the-art approximations we employ. A precise error estimate is difficult to obtain; however, unless *T*_c_ is very low, modern superconductivity methods typically carry an expected uncertainty on the order of 30%^26^. It should also be noted that some studies focus primarily on structural predictions and do not apply sufficient rigor to the prediction of *T*_c_.

## Conclusion

We have investigated whether, within the recently released GNoME dataset of predicted stable (at 0 K) compounds, there is any exceptional hydride superconductor with a high critical temperature. Our high-throughput calculations were able to identify 25 superconductors with *T*c ranging from the boiling point of liquid helium (4.2 K) to 23.5 K. For the highest-*T*_c_ compound, advanced ab initio methods refined *T*_c_ down to 17 K. While not extremely high, as compared to some of the other predictions which have appeared in literature, these stand out because of the extremely accurate estimation of thermodynamic stability at 0K (due to a more densely sampled 0K convex hull). At this stage, experimental feedback that can validate both the crystal structures and superconductivity of these DFT-based candidates would greatly benefit the future search for superconductors.

## Methods

All the ab initio calculations were computed by the Vienna ab initio simulation package (VASP)^59,60^ and Quantum Espresso (QE)^61,62^. In both VASP and Quantum Espresso calculations, the PBEsol functional, a revised version of the Perdew–Burke–Ernzerhof (PBE) generalized gradient approximation (GGA) for solids^63^, was employed for exchange-correlation effects in all the ab initio calculations. In the QE calculations, the PBEsol pseudo potentials from the strict set of PseudoDojo were employed^64^. For elements with *f* electrons, it is noted that they are frozen as the core states in the PseudoDojo potentials.

To prescreen the nonmagnetic and metallic hydrides, spin-polarized density functional theory (DFT) methods implemented in the VASP were used, and those calculations were managed and run by the Quacc code^65^. The kinetic energy cutoff of 550 eV was used for the plane-wave basis. In the structure optimizations, a grid density of 1000 per ${{{{\rm{A}}}}{{{\rm{n}}}}{{{\rm{g}}}}{{{\rm{s}}}}{{{\rm{t}}}}{{{\rm{r}}}}{{{\rm{o}}}}{{{\rm{m}}}}}^{-3}$) of reciprocal cell was used to sample the *k*-grid. Electronic self-consistency was achieved with an energy convergence threshold of 10^−6^ eV. The relaxations were carried out until the forces on all the atoms were smaller than 0.001 eV/Å.

We trained the ALIGNN^28^ model using as targets, simultaneously, *λ*, ${\omega }_{\log }$, and *T*_c_, with the error for each property weighted equally, as these are the choices yielding the best results. We used the default hyperparameters. Data for the training dataset were obtained from refs. ^9,10,66^, and can be downloaded from https://alexandria.icams.rub.de. The used training dataset ensures that all elements are included except the lanthanides and actinides, with La and Lu as the only exceptions. It is noted that the *T*_c_ expands a wide range up to 80 K, providing some coverage of high-*T*_c_ regimes. In addition, all crystal systems except monoclinic and triclinic are represented in the training set, including cubic hydride systems, which are relevant for our target materials in this study.

The other ab initio calculations were all carried out by using Quantum ESPRESSO^61,62^. In the high-throughput Quantum Espresso calculations, geometry optimizations employed uniform Γ-centered *k*-point grids with a density of 3000 *k*-points per reciprocal atom. When this resulted in an odd number of *k*-points in any direction, the next even number was used. Energy, force, and stress convergence thresholds were set to 1 × 10^−8^ a.u., 1 × 10^−6^ a.u., and 5 × 10^−2^ kbar, respectively. For electron–phonon coupling calculations, we utilized a double-grid technique where the *k*-grid from lattice optimization was doubled in each direction for the coarse grid and quadrupled for the fine grid. Phonon *q*-sampling used half of the aforementioned *k*-point grid. The Eliashberg function was obtained through double *δ*-integration using a Methfessel-Paxton smearing of 0.05 Ry.

Ab initio Eliashberg simulations including Coulomb interactions are performed using the approach of ref. ^51^, where Coulomb matrix elements and average Coulomb interaction functions $W(\xi ,\xi ^{\prime} )$ are computed using the elk code^67^. SCDFT anisotropic simulations are done with a Monte-Carlo algorithm using 35 K k-points accumulated with higher probability near the Fermi surface^26,68^. The same algorithm is used to compute the electron-phonon spectral functions for multiband simulations. Electronic and coupling parameters are linearly interpolated on the random mesh, which is used for the solution of the SCDFT Kohn-Sham gap equation. We adopt the function from ref. ^55^. Fermi surfaces are plotted with the Fermisurfer code^49^.

## Supplementary information

Supplementary Information

## Data Availability

The data supporting the findings of this study are included within the article and its Supplementary Information.

## Code availability

The first-principles calculations were performed using the proprietary code VASP^59,60^, and Quantum ESPRESSO^61,62^, which is an open-source suite of computational tools with the GNU General Public License v2.0. The phonon and electron-phonon properties were calculated by Quantum ESPRESSO^61,62^ and SSCHA^32–35^. The SSCHA code (https://github.com/SSCHAcode/python-sscha) is open source and is based on the GNU General Public License v3.0. Quantum ESPRESSO is an open source suite of computational tools with the GNU General Public License v2.0. The crystal structure visualization software VESTA^69^ is distributed free of charge for academic users under the VESTA License (https://jp-minerals.org/vesta/en/).

## References

[1] Drozdov, A. P. et al. Superconductivity at 250 K in lanthanum hydride under high pressures. *Nature* **569**, 528–531 (2019). 31118520 10.1038/s41586-019-1201-8

[2] Somayazulu, M. et al. Evidence for superconductivity above 260 K in lanthanum superhydride at megabar pressures. *Phys. Rev. Lett.* **122**, 027001 (2019). 30720326 10.1103/PhysRevLett.122.027001

[3] Kong, P. et al. Superconductivity up to 243 K in the yttrium-hydrogen system under high pressure. *Nat. Commun.* **12**, 1175 (2021). 34417471 10.1038/s41467-021-25372-2 PMC8379216

[4] Flores-Livas, J. A. et al. A perspective on conventional high-temperature superconductors at high pressure: methods and materials. *Phys. Rep.* **856**, 1–78 (2020).

[5] Boeri, L. et al. The 2021 room-temperature superconductivity roadmap. *J. Phys.* **34**, 183002 (2022). 10.1088/1361-648X/ac2864 34544070

[6] Kolesnikov, A. I. et al. A real-time neutron diffraction study of phase transitions in the Ti-D system after high-pressure treatment. *J. Phys.* **5**, 5045 (1993).

[7] Satterthwaite, C. B. & Toepke, I. L. Superconductivity of hydrides and deuterides of thorium. *Phys. Rev. Lett.* **25**, 741–743 (1970).

[8] Welter, J. M. & Johnen, F. J. Superconducting transition temperature and low temperature resistivity in the niobium-hydrogen system. *Z. Phys. B* **27**, 227–232 (1977).

[9] Cerqueira, T. F. T., Fang, Y.-W., Errea, I., Sanna, A. & Marques, M. A. L. Searching materials space for hydride superconductors at ambient pressure. *Adv. Funct. Mater.* **34**, 2404043 (2024).

[10] Sanna, A. et al. Prediction of ambient pressure conventional superconductivity above 80 k in hydride compounds. *npj Comput. Mater.* **10**, 124 (2024).

[11] Dolui, K. et al. Feasible route to high-temperature ambient-pressure hydride superconductivity. *Phys. Rev. Lett.* **132**, 166001 (2024). 38701475 10.1103/PhysRevLett.132.166001

[12] Tian, C. et al. Few-hydrogen metal-bonded perovskite superconductor MgHCu_3_ with a critical temperature of 42 K under atmospheric pressure. *Adv. Funct. Mater*. **34**, 2304919 (2024).

[13] He, Y., Lu, J., Wang, X. & Shi, J. -J. Phonon-mediated superconductivity in the metal-bonded perovskite Al_4_H up to 54 K under ambient pressure. *Phys. Rev. B* **108**, 054515 (2023).

[14] Zheng, F. et al. Prediction of ambient pressure superconductivity in cubic ternary hydrides with mh6 octahedra. *Mater. Today Phys.* **42**, 101374 (2024).

[15] Gao, K. et al. The maximum *T*_*c*_ of conventional superconductors at ambient pressure. *Nat. Commun.* **16**, 8253 (2025). 40931021 10.1038/s41467-025-63702-w PMC12423309

[16] Li, X. et al. High-tc superconductivity above 130 k in cubic mh4 compounds at ambient pressure. Preprint at *arXiv*10.48550/arXiv.2511.04222 (2025).

[17] Du, M. et al. High-temperature superconductivity in perovskite hydride below 10 GPa. *Adv. Sci.* **11**, 2408370 (2024). 10.1002/advs.202408370 PMC11558092 39301938

[18] He, Y. et al. Metal-bonded perovskite lead hydride with phonon-mediated superconductivity exceeding 46 k under ambient pressure. *Phys. Condens. Matter* **36**, 205502 (2024). 10.1088/1361-648X/ad2806 38335547

[19] Dangić, D. et al. Ambient pressure high temperature superconductivity in RbPH3 facilitated by ionic anharmonicity. *Comput. Mater. Today* **8**, 100043 (2025).

[20] Cerqueira, T. F. T., Sanna, A. & Marques, M. A. L. Sampling the whole materials space for conventional superconducting materials. Preprint at *Adv. Mater.* **36**, 2307085 (2024). 10.1002/adma.202307085 37985412

[21] Deng, L. et al. Pressure-induced high-temperature superconductivity retained without pressure in fese single crystals. *Proc. Natl. Acad. Sci. USA* **118**, e2108938118 (2021). 34234019 10.1073/pnas.2108938118 PMC8285973

[22] Merchant, A. et al. Scaling deep learning for materials discovery. *Nature* **624**, 80–85 (2023). 10.1038/s41586-023-06735-9 PMC10700131 38030720

[23] Wang, H.-C., Botti, S. & Marques, M. A. Predicting stable crystalline compounds using chemical similarity. *npj Comput. Mater.* **7**, 12 (2021).

[24] Kirklin, S. et al. The open quantum materials database (oqmd): assessing the accuracy of DFT formation energies. *npj Comput. Mater.* **1**, 864 (2015).

[25] Chen, C. & Ong, S. P. A universal graph deep learning interatomic potential for the periodic table. *Nat. Comput. Sci.* **2**, 718–728 (2022). 38177366 10.1038/s43588-022-00349-3

[26] Pellegrini, C. & Sanna, A. Ab initio methods for superconductivity. *Nat. Rev. Phys.* **6**, 509–523 (2024).

[27] *GNoME Database*. https://github.com/google-deepmind/materials_discovery.

[28] Choudhary, K. & DeCost, B. Atomistic line graph neural network for improved materials property predictions. *npj Comput. Mater*. **7**, 185 (2021).

[29] Hilleke, K. P. et al. Structure, stability, and superconductivity of n-doped lutetium hydrides at kbar pressures. *Phys. Rev. B* **108**, 014511 (2023).

[30] Ferreira, P. P. et al. Search for ambient superconductivity in the Lu-N-H system. *Nat. Commun.* **14**, 244 (2023). 37666834 10.1038/s41467-023-41005-2 PMC10477194

[31] Errea, I. et al. Quantum crystal structure in the 250-kelvin superconducting lanthanum hydride. *Nature* **578**, 66–69 (2020). 32025016 10.1038/s41586-020-1955-z

[32] Errea, I., Calandra, M. & Mauri, F. Anharmonic free energies and phonon dispersions from the stochastic self-consistent harmonic approximation: application to platinum and palladium hydrides. *Phys. Rev. B* **89**, 064302 (2014).

[33] Monacelli, L. et al. The stochastic self-consistent harmonic approximation: calculating vibrational properties of materials with full quantum and anharmonic effects. *J. Phys.* **33**, 363001 (2021). 10.1088/1361-648X/ac066b 34049302

[34] Bianco, R., Errea, I., Paulatto, L., Calandra, M. & Mauri, F. Second-order structural phase transitions, free energy curvature, and temperature-dependent anharmonic phonons in the self-consistent harmonic approximation: Theory and stochastic implementation. *Phys. Rev. B* **96**, 014111 (2017).

[35] Monacelli, L., Errea, I., Calandra, M. & Mauri, F. Pressure and stress tensor of complex anharmonic crystals within the stochastic self-consistent harmonic approximation. *Phys. Rev. B* **98**, 024106 (2018).

[36] Allen, P. B. & Dynes, R. C. Transition temperature of strong-coupled superconductors reanalyzed. *Phys. Rev. B* **12**, 905–922 (1975).

[37] Golubov, A. A. et al. Specific heat of mgb2 in a one- and a two-band model from first-principles calculations. *J. Phys.* **14**, 1353 (2002).

[38] Mazin, I. & Antropov, V. Electronic structure, electron-phonon coupling, and multiband effects in MgB2. *Phys. C* **385**, 49–65 (2003).

[39] Scalapino, D. J., Schrieffer, J. R. & Wilkins, J. W. Strong-coupling superconductivity. I. *Phys. Rev.* **148**, 263–279 (1966).

[40] Massidda, S. et al. The role of coulomb interaction in the superconducting properties of cac 6 and h under pressure. *Supercond. Sci. Technol.* **22**, 034006 (2009).

[41] Marini, G. et al. Superconducting Chevrel phase PbMo_6_S_8_ from first principles. *Phys. Rev. B* **103**, 144507 (2021).

[42] Morel, P. & Anderson, P. W. Calculation of the superconducting state parameters with retarded electron-phonon interaction. *Phys. Rev.* **125**, 1263–1271 (1962).

[43] Rietschel, H. & Winter, H. Role of spin fluctuations in the superconductors Nb and V. *Phys. Rev. Lett.* **43**, 1256–1260 (1979).

[44] Tsutsumi, K., Hizume, Y., Kawamura, M., Akashi, R. & Tsuneyuki, S. Effect of spin fluctuations on superconductivity in V and Nb: a first-principles study. *Phys. Rev. B* **102**, 214515 (2020).

[45] Pellegrini, C., Kukkonen, C. & Sanna, A. Ab initio calculations of superconducting transition temperatures: when going beyond RPA is essential. *Phys. Rev. B* **108**, 064511 (2023).

[46] Suhl, H., Matthias, B. & Walker, L. Bardeen-Cooper-Schrieffer theory of superconductivity in the case of overlapping bands. *Phys. Rev. Lett.* **3**, 552–554 (1959).

[47] Floris, A. et al. Superconducting properties of MgB_2_ from first principles. *Phys. Rev. Lett.* **94**, 037004 (2005). 15698310 10.1103/PhysRevLett.94.037004

[48] Floris, A. et al. Superconducting properties of MgB_2_ from first principles. *Physica C* **456**, 45–53 (2007). 10.1103/PhysRevLett.94.037004 15698310

[49] Kawamura, M. Fermisurfer: Fermi-surface viewer providing multiple representation schemes. *Comput. Phys. Commun.* **239**, 197–203 (2019).

[50] Sanna, A. et al. Ab initio Eliashberg theory: making genuine predictions of superconducting features. *J. Phys. Soc. Jpn.* **87**, 041012 (2018).

[51] Pellegrini, C., Heid, R. & Sanna, A. Eliashberg theory with ab-initio Coulomb interactions: a minimal numerical scheme applied to layered superconductors. *J. Phys.* **5**, 024007 (2022).

[52] Essenberger, F. et al. Superconducting pairing mediated by spin fluctuations from first principles. *Phys. Rev. B* **90**, 214504 (2014).

[53] Essenberger, F. et al. Ab initio theory of iron-based superconductors. *Phys. Rev. B* **94**, 014503 (2016).

[54] Kukkonen, C. A. & Overhauser, A. W. Electron-electron interaction in simple metals. *Phys. Rev. B* **20**, 550–557 (1979).

[55] Sanna, A., Pellegrini, C. & Gross, E. K. U. Combining Eliashberg theory with density functional theory for the accurate prediction of superconducting transition temperatures and gap functions. *Phys. Rev. Lett.* **125**, 057001 (2020). 32794891 10.1103/PhysRevLett.125.057001

[56] Wang, X., Pickett, W. E., Hutcheon, M., Prasankumar, R. P. & Zurek, E. Why Mg2IrH6 is predicted to be a high-temperature superconductor, but Ca2IrH6 is not. *Angew. Chem. Int. Ed.* **63**, e202412687 (2024). 10.1002/anie.202412687 39393009

[57] Hansen, M. F. et al. Synthesis of Mg2IrH5: A potential pathway to high-*T*_*c*_ hydride superconductivity at ambient pressure. *Phys. Rev. B* **110**, 214513 (2024).

[58] Wang, A. et al. A framework for quantifying uncertainty in DFT energy corrections. *Sci. Rep.* **11**, 438 (2021). 34326361 10.1038/s41598-021-94550-5 PMC8322326

[59] Kresse, G. & Furthmüller, J. Efficiency of ab-initio total energy calculations for metals and semiconductors using a plane-wave basis set. *Comput. Mater. Sci.* **6**, 15–50 (1996). 10.1103/physrevb.54.11169 9984901

[60] Kresse, G. & Furthmüller, J. Efficient iterative schemes for ab initio total-energy calculations using a plane-wave basis set. *Phys. Rev. B* **54**, 11169–11186 (1996). 10.1103/physrevb.54.11169 9984901

[61] Giannozzi, P. et al. Quantum espresso: a modular and open-source software project for quantum simulations of materials. *J. Phys.* **21**, 395502 (2009). 10.1088/0953-8984/21/39/395502 21832390

[62] Giannozzi, P. et al. Advanced capabilities for materials modelling with Quantum Espresso. *J. Phys.* **29**, 465901 (2017). 10.1088/1361-648X/aa8f79 29064822

[63] Perdew, J. P. et al. Restoring the density-gradient expansion for exchange in solids and surfaces. *Phys. Rev. Lett.* **100**, 136406 (2008). 18517979 10.1103/PhysRevLett.100.136406

[64] van Setten, M. et al. The PseudoDojo: training and grading a 85 element optimized norm-conserving pseudopotential table. *Comput. Phys. Commun.* **226**, 39–54 (2018).

[65] Rosen, A. Quacc—the Quantum Accelerator. *Zenodo*. 10.5281/zenodo.13139853 (2024).

[66] Cerqueira, T. F. T., Sanna, A. & Marques, M. A. L. Sampling the materials space for conventional superconducting compounds. *Adv. Mater.* **36**, 2307085 (2024). 10.1002/adma.202307085 37985412

[67] *The Elk FP-LAPW Code*. https://elk.sourceforge.net.

[68] Sanna, A. et al. Real-space anisotropy of the superconducting gap in the charge-density wave material 2H-NbSe2. *npj Quantum Mater.* **7**, 6 (2022).

[69] Momma, K. & Izumi, F. VESTA 3 for three-dimensional visualization of crystal, volumetric and morphology data. *J. Appl. Crystallogr.* **44**, 1272–1276 (2011).

