# Supplementary material for: Search for thermodynamically stable ambient-pressure superconducting hydrides in the GNoME database
Source: Commun Phys. 2026 Feb 27;9(1):94. doi: 10.1038/s42005-026-02552-4 (PMC12992110; doi:10.1038/s42005-026-02552-4)
Supplement: Supplementary file 1 — Supplementary Information [file 42005_2026_2552_MOESM1_ESM.pdf]

Supplementary Information for " Search for thermodynamically stable ambient-pressure superconducting hydrides in GNoME database "

Antonio Sanna§,[1,2,*] Tiago F. T. Cerqueira§,[3] Ekin Dogus Cubuk,[4] Ion Errea,[5,6,7,†] and Yue-Wen Fang[6,‡]

[1] *Max-Planck-Institut für Mikrostrukturphysik, Weinberg 2, D-06120 Halle, Germany*
[2] *Institut für Physik, Martin-Luther-Universität Halle-Wittenberg, D-06099 Halle, Germany*
[3] *CFisUC, Department of Physics, University of Coimbra, Rua Larga, 3004-516 Coimbra, Portugal*
[4] *No current affiliation*
[5] *Fisika Aplikatua Saila, Gipuzkoako Ingeniaritza Eskola, University of the Basque Country (UPV/EHU), Europa Plaza 1, 20018 Donostia/San Sebastián, Spain*
[6] *Centro de Física de Materiales (CFM-MPC), CSIC-UPV/EHU, Manuel de Lardizabal Pasealekua 5, 20018 Donostia/San Sebastián, Spain*
[7] *Donostia International Physics Center (DIPC), Manuel de Lardizabal Pasealekua 4, 20018 Donostia/San Sebastián, Spain*

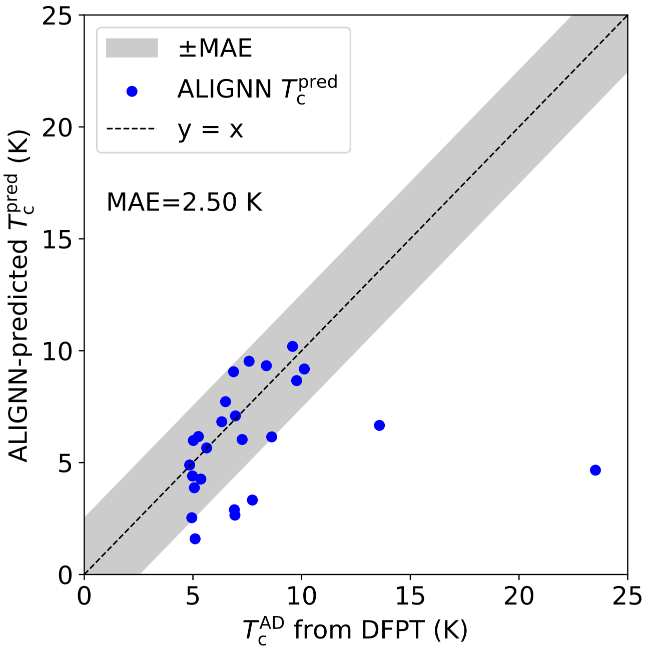

FIG. S1. The comparison between ALIGNN predicted T_c^{pred} and the DFPT computed T_c^{AD} for the 25 identified cubic hydride superconductors.

* sanna@mpi-halle.mpg.de
† ion.errea@ehu.eus
‡ yuewen.fang@ehu.eus

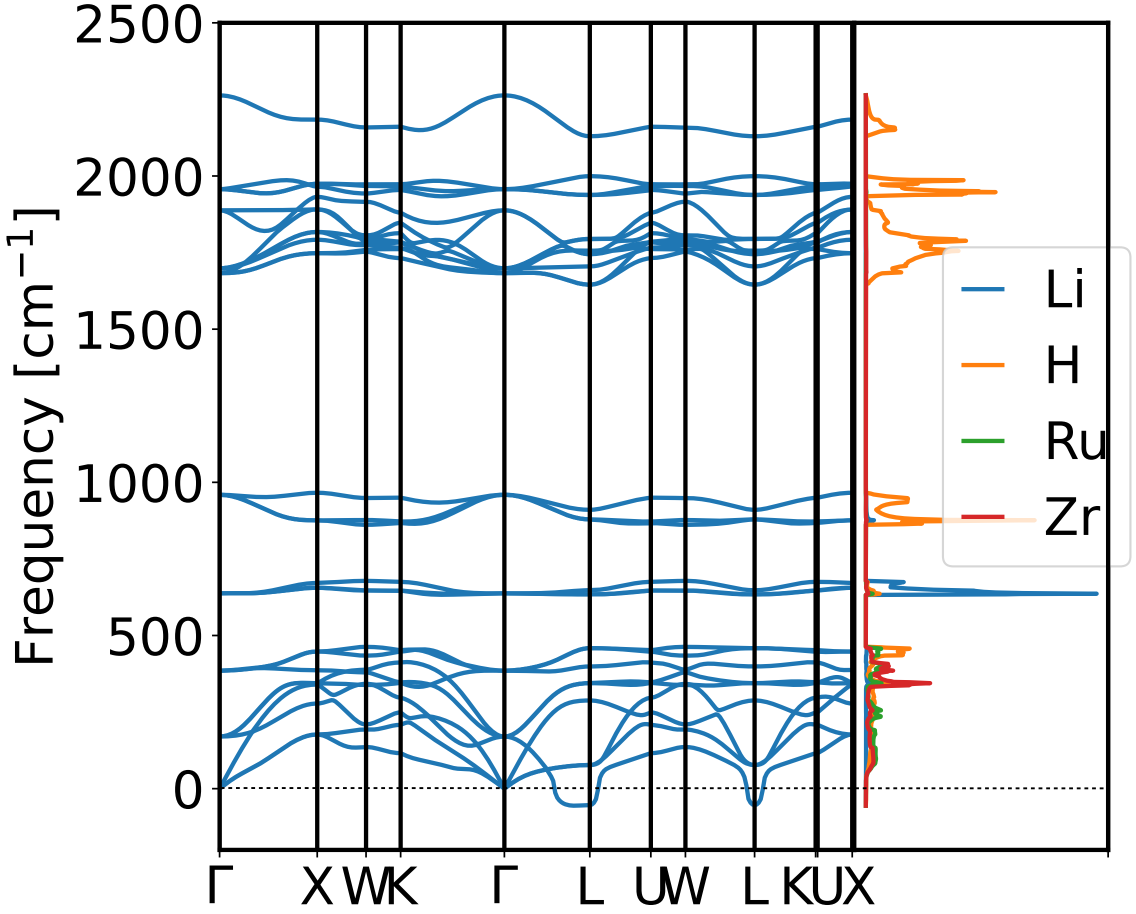

FIG. S2. Phonon properties of $LiZrH_6Ru$ at 200 GPa in the harmonic approximation. The L point shows imaginary modes.

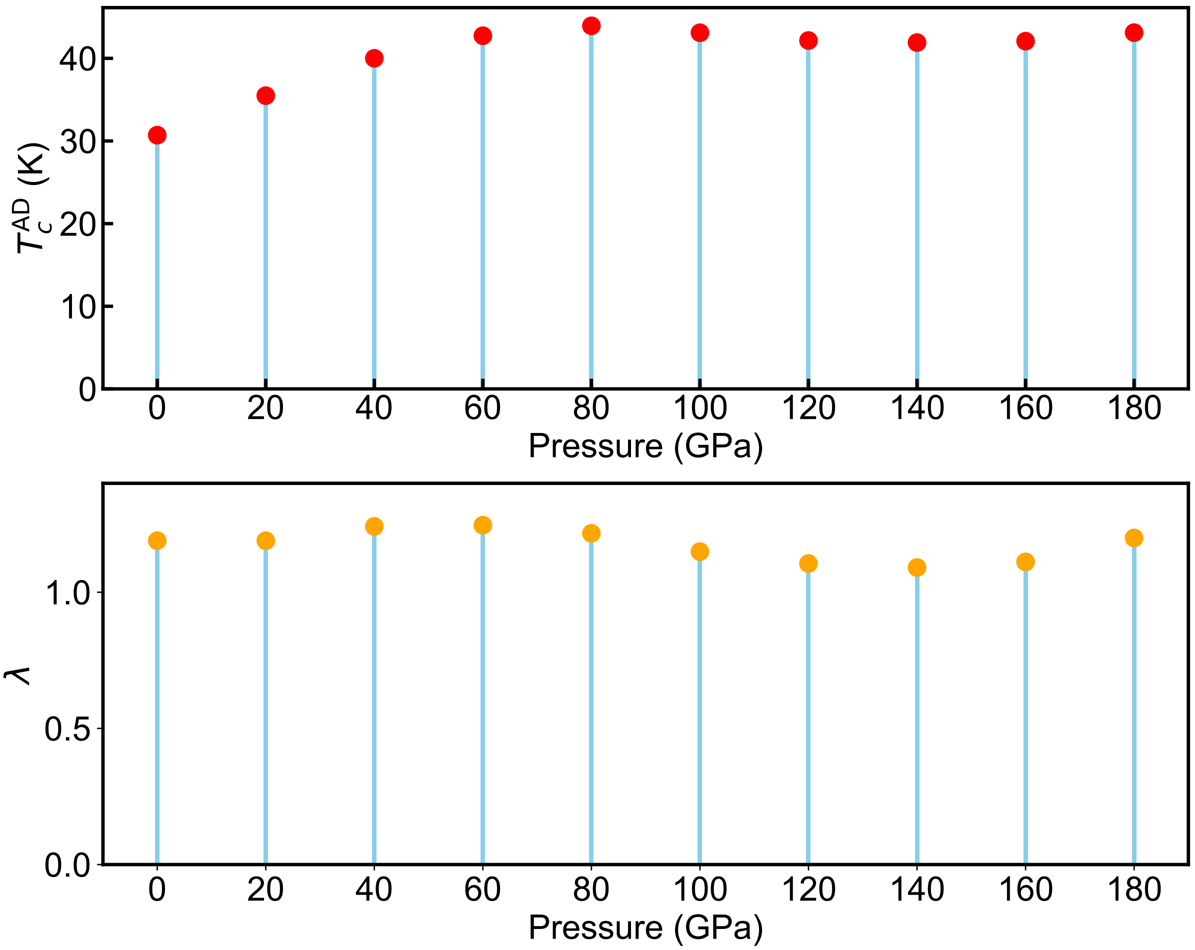

FIG. S3. The evolution of T_c^{AD} and electron-phonon coupling constant λ at pressures in $LiZrH_6Ru$.
